# Immediate postpartum anemia and associated factors at shewarobit health facilities, Amhara, Ethiopia, 2022: a cross sectional study

**DOI:** 10.1186/s12905-024-03017-y

**Published:** 2024-03-20

**Authors:** Nigus Amime Eshete, Yohannes Moges Mittiku, Alemayehu Gonie Mekonnen, Tesfay Hailu Welu, Teklehaimanot Gereziher Haile

**Affiliations:** 1 Department of Midwifery, Shewarobit Hospital, North Shewa, Amhara, Ethiopia; 2 Department of Midwifery, College of Health Sciences, Debre Brhan University, Debre Brhan, Ethiopia; 3 School of Nursing and Midwifery, College of Health Sciences, Debre Brhan University, Debre Brhan, Ethiopia; 4 Department of Midwifery, College of Health Sciences and Comprehensive Specialized Hospital, Aksum University, Aksum, Tigray, Ethiopia; 5 Department of Maternity and Neonatal Nursing, School of Nursing, College of Health Sciences, Comprehensive Specialized Hospital, Aksum University, Aksum, Tigray, Ethiopia

**Keywords:** Amhara, Anemia, Associated factors, Ethiopia, Immediate postpartum, Mothers, Proportion

## Abstract

**Background:**

Immediate postpartum anemia occurs when the amount of red blood cell count is reduced or hemoglobin concentration is below 10 g/dl in the immediate postpartum. It occurs primarily due to inadequate iron intake before and during pregnancy and blood loss during delivery. The aim of this study is to assess the proportion of immediate postpartum anemia and associated factors among mothers who gave birth at Shewarobit health facilities; in Amhara, Ethiopia.

**Methods:**

Institutional-based cross-sectional study was conducted from June to September 2022. A systematic random sampling method was employed to select the study participants. The data were collected through interviewer-assisted questions. Data were entered into Epi Data software version 4.6.0.4 and exported to SPSS 21 for analysis, and descriptive statistics were computed. Logistic regression was applied, and P-values less than 0.05 were considered statistically significant.

**Results:**

This study was conducted among 307 study participants and, the proportion of immediate postpartum anemia was 41.4% [95% CI: 36.7–46.6]. Having postpartum hemorrhage [AOR = 4.76, 95% CI: 2.44–9.28], not taking iron and folic acid supplementation [AOR = 6.19, 95% CI: 2.69, 14.22], having a prolonged second stage of labor [AOR = 2.52, 95% CI: 1.16–5.44], and mid-upper arm circumference < 23 cm [AOR = 2.02, 95% CI: 1.11–3.68] were factors significantly associated with immediate postpartum anemia.

**Conclusions:**

The proportion of immediate postpartum anemia was public problem in Shewarobit health facilities. Following the progress of labor using a partograph, closely monitoring and immediate intervention of PPH, and prevent undernutrition during antenatal care is recommended.

## Background

Postpartum anemia continues to be a major public health concern in developing nations, especially in sub-Saharan Africa. It is the most frequent non-direct cause of death and morbidity in mothers [1]. Immediate postpartum anemia is defined as Hgb < 10 g/dl, Hgb < 11 g/dl, and Hgb < 12 g/dl cut-off values within the first 48 h of delivery, at 1 week and 6 weeks of postpartum duration, respectively [1–4]. Immediate postpartum anemia could be defined as postpartum anemia within 24 h of the delivery of the placenta [5]. It is also known as the postnatal period, or puerperium [6–9]. Similarly, the immediate postpartum period refers to the time just after delivery of the placenta, during which the risks of postpartum hemorrhage and other significant morbidity are highest and covers the first 24 h from birth [9].

Immediate Post-Partum Anemia (IPPA) occurs when the red blood cell count is reduced or the hemoglobin concentration is below 10 g/dl in the immediate postpartum [10–13]. , primarily due to inadequate iron intake before and during pregnancy and blood loss during delivery. In other words, the combination of iron deficiency anemia (IDA) and hemorrhagic anemia leads to postpartum anemia [14]. The magnitude of postpartum hemorrhage in Ethiopia was 8.18%, which is a leading direct cause of maternal morbidity and mortality [14]. IDA is common during the postpartum period and accounts for 50% of cases of anemia [15, 16].

Immediate postpartum anemia is a global health problem in both developing and developed countries, with major consequences for maternal health as well as social and economic development [4]. Worldwide, the World Health Organization (WHO) estimated the number of anemic mothers to be about 1.5 billion, and approximately 50% of all cases can be attributed to iron deficiency [5, 17]. It is estimated that 1 out of 5 maternal deaths are caused by postpartum hemorrhage and anemia [6–8]. In 2017, 295 000 women died during and following pregnancy and childbirth. The vast majority occurred in low-resource settings [18].

Although the incidence of postpartum anemia is estimated to be low in countries with high development, in countries with low development, the incidence is as high as 50–80% [10]. The most at-risk population groups are both pregnant women and women of reproductive age, next to children, for anemia on a global scale [11]. African and Asian countries account for more than 80% of the anemia burden in high-risk groups [12].

In Ethiopia, anemia accounted for 10.39% of all reported indirect causes of maternal mortality. Therefore, the maternity ward presents a crucial window of opportunity for postpartum women to get medical management for anemia [19]. The Ministry of Health has advocated nutritional treatments, such as the Essential Nutrition Action Plan, which consists of vitamin A, iron, iodine supplements and, others to lessen the incidence of anemia among Ethiopian women [20].

Anemia associated with infections has also contributed to postpartum anemia [5]. According to WHO, pregnancy-specific hemoglobin levels are used to categorize anemia in pregnancy and the postpartum period; that is, 10–10.9 g/dl is considered mild anemia, 7–9.9 g/dl is considered moderate, and < 7 g/dl is considered severe anemia [5, 21, 22]. When the hemoglobin concentration falls below 4.1 g/dl most of the body tissues become starved of oxygen, and the heart muscles are likely to fail, resulting in death [10, 23].

Despite the fact that studies on anemia have been conducted in the general population, there is insufficient evidence on the proportion and factors associated with immediate postpartum anemia among mothers who gave birth in Shewarobit health facilities. Therefore, the aim of this study is to assess the proportion of immediate postpartum anemia and associated factors among mothers who gave birth at Shewarobit health facilities; in Amhara, Ethiopia. As a result, postpartum mothers may benefit from the intervention taken based on result findings. This study might also provide insight into IPPA for midwives as a baseline in their counseling to minimize IPPA. Furthermore, this might help stakeholders and policymakers to strengthen their plans for the prevention of immediate postpartum anemia.

## Methods

### Study design, period, and study setting

A facility-based cross-sectional study was conducted from June to September 2022 at Shewarobit health facilities. This is located in the North Shewa Zone, Amhara Region, Ethiopia. It has nine administrative units (Kebele) with a total population of 50,528, of which 25,890 (51.2%) are women.

### Source and study population

The source population consisted of all postpartum mothers who gave birth at Shewarobit Health Facilities as well as mothers who gave birth elsewhere but visited the hospital within 24 h of the postpartum period. The study population consisted of mothers who met the requirements to be a source population and made themselves available during the data collection period.

### Inclusion and exclusion criteria

This study included all postnatal women who gave birth at Shewarobit Health Facilities as well as mothers who gave birth elsewhere but visited the hospital within a day of the data collection period. This study did not include mothers who were anemic prior to conception or during pregnancy.

### Sample size determination and sampling procedure

The sample size calculation was based on the single population proportion formula by using the following assumptions: proportion = 24.3% from previous studies [5], z = standard normal distribution at 95% confidence interval (1.96), α = level of significance (5%), d = margin of error (5%), n = required sample size, then the formula for calculating the sample size is,

n = (z_α/2_)^2^× P (1-P) = (1.96)^2^ × 0.243(1-0.243) therefore, *n* = 283.

d^2^ (0.05)^2^

Epi Info version 7.2.5.0 was used to determine the sample size based on the second objective, which is statistically significant factors. A maximum sample size of 198 was determined. As a result, the sample size indicated by the second objective was smaller than the one computed by the first objective. Therefore, the final sample size was determined using the sample size derived from the first objective. The bare minimum suitable sample size, considering a 10% non-response rate, was 311. Based on the high number of maternity ward admission services provided by these three public institutions, the sample size was proportionately assigned to each, considering the average monthly maternity admission from each facility’s health management and information system report.

### Study variables

#### Dependent variables: immediate postpartum anemia

Independent variables: sociodemographic characteristics, obstetrical-related variables, Coexisting infection-related variables, and dietary and micronutrient uptake-related variables.

### Data Collection Tools and procedures

Data were collected using an interviewer assigned questionnaire, which was developed after reviewing different pieces of literature [5, 17, 24, 25] conducted in Ethiopia and in different parts of the world, the questionnaire contains sociodemographic characteristics, obstetrical-related variables, coexisting infection-related variables, and dietary and micronutrient-uptake-related variables. A total of four BSc midwives were recruited for data collection and supervision. Data were collected through a face-to-face interview and supplemented by a maternal chart review. MUAC was measured via tape meters on the non-dominant hand, mostly the left hand. The result was interpreted according to WHO recommendations of a cutoff point of < 23 cm as undernourished and ≥ 23 cm as well-nourished.

### Laboratory procedures

The 3 health facilities used the “HORIBA ABX Micros 60 Hematology Analyzer.” For quality control, a sample of blood was taken and tested in all laboratories of health facilities, and the hemoglobin level was the same. To estimate hemoglobin concentration, about 4 milliliters of anticoagulated sample blood from a superficial vein on the forearm were drawn into an ethylene diamine tetra acetic acid (EDTA) vacationer by data collectors and taken to respective laboratories. The laboratory technologist measured the hemoglobin level using an automated hematology analyzer. Using a closed mode of blood sampling, the analyzer automatically sampled, processed, and analyzed blood and printed out hemoglobin levels. Finally, the levels of hemoglobin were attached to their respective charts. At the end, anemic mothers were treated with iron and folic acid or transfused with blood based on their hemoglobin levels and were advised on iron-rich diets.

### Data quality control management

In order to assure data quality, the English version of the questioner was translated into Amharic, and to keep its consistency, it was translated back to the English version. A pretest was done on 16 immediate postpartum mothers at Ataye Primary Hospital before the actual data collection. This helps to check the response, language clarity, and appropriateness of the questionnaire. Also, one-day training was given to data collectors and supervisors about the purpose of the study and the procedures for data collection. Finally, the data were checked on a daily basis during data collection periods by the supervisor to confirm its completeness and the presence of missing data.

### Data processing and analysis

After data collection was completed, information was assessed for its completeness, numerical coding was implemented, and it was entered into Epi Data Software version 4.6.0.4, and exported to Statistical Package for Social Science (SPSS) version 21 software for further analysis. Descriptive statistics were computed and presented with texts, tables, and graphs. Cross-tabulation and Chi-square assumptions were checked. A binary logistic regression model was fitted to identify independent factors, and variables having a p-value of less than 0.2 were entered into the multivariable logistic regression to handle possible confounders, and the crude odds ratio (COR) was computed. In the multivariable logistic regression, a p-value of ≤ 0.05 with a 95% CI for the adjusted odds ratio (AOR) was used to affirm the statistical association.

## Results

### Sociodemographic characteristics

Three hundred seven immediate postnatal mothers were involved in this study, giving a total response rate of about 98.7%. The minimum age was 17 and the maximum was 45, and the mean age of the study population was 26.92 ± SD 5.34 years. Based on the age category, 145 (47.2%) study participants were in the age range of 25–34 years. About 60 (19.5%) of mothers were unable to read and write, and 147 (47.9%) of mothers were housewives by their occupation. About 198 (64.5%) were orthodox Christian followers. More than half (213), or 69.4%of the participants were from urban areas. Regarding marital status, 98% (301) of women were married. About one-third (32.6%) of husbands were farmers (Table 1).

**Table 1:** Sociodemographic characteristics of postpartum mothers in Shewarobit health facilities (*n* = 307)

Variable	Category	Frequency (n)	percent (%)
Age	15–24 25–34 35–49	130 145 32	42.3 47.2 10.4
Religion	Orthodox Muslim Protestant	198 86 23	64.5 28 7.5
Residency	Urban Rural	213 94	69.4 30.6
Ethnic Group	Amhara Afar Argoba Oromo Tigre	245 17 19 18 8	79.8 5.5 6.2 5.9 2.6
Educational Status	Un able to read and write Able to read and write Primary class completed Secondary class completed Diploma and above	60 86 58 66 37	19.5 28 18.9 21.5 12.1
Occupation	House Wife Government Employee Private Employee Merchant Farmer	147 34 40 36 50	47.9 11.1 13 11.7 16.3
Marital Status	Married Un Married	301 6	98 2
Monthly Income (ET birr)	< 2000 2000–5000 5001–10,000 ≥ 10,001	34 163 98 12	11.1 53.1 31.9 3.9

### Obstetrical related factors

Among the total 307 study participants, 106 (34.5%) were primipara mothers. A majority of 287 (93.5%) of the study participants had antenatal care follow-up during their recent pregnancy. From those, more than half 190 (61.9%) of the mothers had ≥ 4 ANC visits. Sixty-one participants gave birth before 37 weeks of gestation, and sixty mothers faced a prolonged second stage of labor. From the total participants, 212 gave birth by SVD, whereas 51 (16.6%) of the study participants gave birth through a cesarean section, and 72 participants experienced PPH in their immediate postpartum period (Table 2).

**Table 2:** Obstetrical characteristics of postnatal mothers in Shewarobit health facilities, North Shewa, Ethiopia 2022 (*n* = 307)

Variable	Category	Frequency (n)	Percent (%)
Parity	Primi Para Multi Parous	106 201	34.5 65.5
Inter pregnancy Interval of the last and present (in months)	< 24 ≥ 24	39 162	12.7 52.8
Twin Pregnancy	Yes No	12 295	3.9 96.1
APH	Yes No	29 278	9.4 90.6
ANC	Yes No	287 20	93.5 6.5
#of ANC	< 4 ≥ 4	97 190	31.6 61.9
GA at first ANC (in weeks)	< 16 ≥ 16	160 127	52.1 41.4
GA at delivery	< 37 ≥ 37	61 246	19.9 80.1
Mode of Delivery	SVD IAVD C/S	212 44 51	69.1 14.3 16.6
Prolonged second stage	Yes No	60 247	19.5 80.5
Episiotomy	Yes No	85 222	27.7 72.3
Perineal Tear	Yes No	54 253	17.6 82.4
PPH	Yes No	72 235	23.5 76.5
New Born Birth Weight(gm)	< 4000 ≥ 4000	299 8	97.4 2.6

### Coexisting infection-related factors

Forty-two mothers were dewormed with mebendazole, and 129 mothers used ITN for the prevention of helminthes and malaria, respectively. One hundred sixty-five (53.7%) of the respondents had diagnosed coexisting infections during recent pregnancy, of which UTI were predominant (11.4%) whereas syphilis (1.6%) was the least (Table 3).

**Table 3:** Coexisting infections related factors among postnatal mothers in Shewarobit health facilities 2022 (*n* = 307)

Variable	Category	Frequency	Percent (%)
De-wormed with Mebendazole	Yes No	42 265	13.7 86.3
Helminthes during pregnancy	Yes No	32 275	10.4 89.6
ITN used	Yes No	129 178	42 58
Malaria positive	Yes No	7 300	2.3 97.7
HIV/AIDS	Yes No	13 294	4.2 95.8
Syphilis positive	Yes No	5 302	1.6 98.4
UTI positive	Yes No	35 272	11.4 88.6

Note: Type of helminthes were Ameobiasis, Giardiasis and Shcistosomiasis

### Dietary and micronutrients utilization related factors

Two hundred sixty-five of the study participants were started on IFA tablets during recent pregnancy. Among these, 149 (48.5%) were started before 20 weeks of gestation. Among mothers supplied with IFA tablets, 216 (70.4%) took their iron supplementation for at least 90 days. Ninety-four (30.6%) of the mothers’ mid-upper arm circumference was less than 23 cm (Table 4).

**Table 4:** dietary and micronutrient related factors of immediate postpartum anemia in Shewarobit health facilities (*n* = 307)

Variable	Category	Frequency	Percent (%)
Iron taken	Yes No	265 42	86.3 13.7
GA of Iron initiated (weeks)	< 20 weeks ≥ 20 weeks	149 116	48.5 37.8
Duration of Iron supply	< 3 months ≥ 3 months	49 216	16.0 70.4
MUAC (cm)	< 23 ≥ 23	94 213	30.6 69.4
Hgb determination time	< 8 h	244	79.5
	≥ 8 h	63	20.5

### The proportion of Immediate Postpartum Anemia

**Fig. 1:**
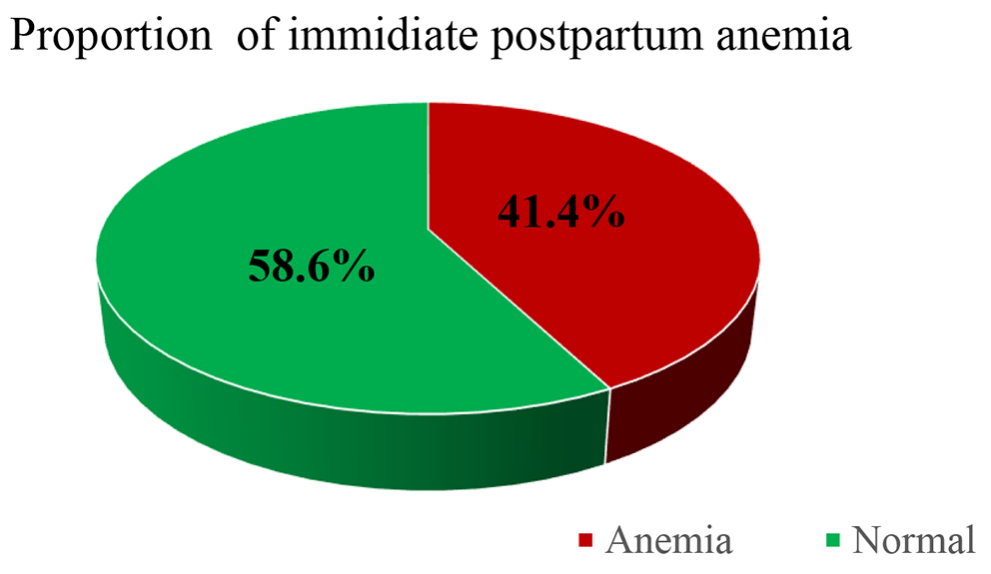
proportion of immediate postpartum anemia among mothers who gave birth at Shewarobit health facilities, Amhara, Ethiopia, 2022(*n* = 307)

Immediate postpartum anemia was observed among 127 (41.4%) mothers. The postpartum hemoglobin concentrations of study participants ranged from 2 g/dl to 17 g/dl, with a mean value of 11.56 g/dl, and SD ± 1.755 g/dl. From the total 41.4% of anemic mothers, 87 (28.3%), 36 (11.7%), and 4 (1.3%) of them were categorized as having mild, moderate, and/or severe anemia, respectively. Regarding hemoglobin determination, the time from delivery to sample collection was recorded in hours, and the mean time of sample collection was 6:17, the minimum time was 1:00, and the maximum time was 22:00, and range of 21:00 (Fig. 1).

### Associated factors of Immediate Postpartum Anemia

In bi-variable logistic regression analysis, maternal educational level (being unable to read and write), preterm delivery, prolonged second stage of labor, perineal tear, postpartum hemorrhage, not taking IFA supplementation, cesarean section mode of delivery, rural residency, and MUAC < 23 cm were variables associated with immediate PPA with a p value of < 0.2. Postpartum hemorrhage, not using IFA supplements, protracted second stage of labor, and MUAC < 23 cm were independent variables in multivariate logistic regression that were substantially associated with a p value < 0.05 (Table 5).

**Table 5:** Multivariable Logistic regression showing associated factors of immediate postpartum anemia among mothers in Shewarobit health facilities, Amhara, Ethiopia, 2022 (*n* = 307)

Variables	Categories	Postpartum anemia		COR 95%CI	AOR 95%CI
		Yes	No		
Educational status	Un able to read and write	36	24	2.46(0.17–7.94)	0.41(0.14–1.18)
	able to read and write	37	49	1.24(0.37–3.78)	0.71(0.27–1.88)
	Primary school completed	19	39	0.8(0.53–1.96)	0.69(0.25–1.93)
	Secondary school completed	21	45	0.77(0.56–1.03)	0.92(0.33–2.54)
	Diploma and above	14	23	1	1
PPH	Yes	49	23	4.29(2.44–7.54)	4.76(2.44–9.28)**
	No	78	157	1	1
Prolonged second stage	Yes	39	21	3.36(1.86–6.06)	2.52(1.16–5.44)*
	No	88	159	1	1
IFA intake status	No	32	10	5.73(2.70-12.16)	6.19(2.69–14.22)**
	Yes	95	170	1	
MUAC	< 23 cm	49	45	1.88(1.15–3.08)	2.02(1.11–3.68)*
	≥ 23 cm	78	135	1	1
GA at delivery	Preterm	33	28	1.91(1.08–3.36)	1.81(0.92–3.54)
	Term	94	152	1	1
Perineal tear	Yes	30	24	2.01(1.11–3.64)	1.82(0.90–3.70)
	No	97	156	1	1
Mode of delivery	C/S	26	25	1.71(0.32–3.08)	0.50(0.16–1.63)
	IAVD	21	23	1.51(0.34–2.28)	0.98(0.41–2.32)
	SVD	80	132	1	1
Residency	Rural	45	49	1.47(0.90–2.39)	1.14(0.60–2.14)
	Urban	82	131	1	1

Note: Reference category * p value less than 0.05 **p value less than 0.01, CI = Confidence interval COR = Odds Ratio, AOR = Adjusted Odds Ratio

## Discussion

This study assessed the proportion and associated factors of immediate postpartum anemia among mothers in the immediate postpartum period in Shewarobit health facilities in 24 h. The proportion of immediate postpartum anemia among study participants was 41.4% (hemoglobin level below 11 g/dl), with a 95% CI: (36.7–46.6), which is slightly higher than the study conducted in China and Tanzania, in which (32.7%) [26] and 34.2% [17] postpartum mothers were found to be anemic, respectively. The proportion of this study was significantly higher than the study conducted in Costal Karnataka, the Ethiopian National Demographic Health Survey (EDHS 2016), and the study conducted in Mekele and DebreMarkos, where 26.5% [27], 24% [14], 24.2% [10], and 24.3% [5] mothers were identified as being anemic in their postpartum period, respectively.

However, the proportion was significantly lower than findings from Indonesia, Myanmar, and Burkina Faso, where 60%, 73.8%, and 51.9% (more than half of their study participants) were anemic [10, 16, 28]. The proportion was slightly lower than the study conducted in India (47.3%), Turkey (45.11%), Spain (45%), and Pakistan (47.9%) [28–31], respectively. The mean hemoglobin concentration (11.56 g/dl) was also lower than the study conducted at DebreMarkos (12.4 g/dl) [5]. This might be due to the geographical differences and socio-cultural factors of Ethiopians, as most mothers take longer maternity rest before child delivery and are kept nourished with a variety of foods. In addition, the majority of Ethiopians consume injera, which is rich in dietary iron. The recentness of this study might also be another factor due to the increased awareness of postpartum mothers about postpartum anemia.

Regarding the severity of anemia, 1.3% of the study participants were severely anemic hemoglobin concentration less than 7 g/dl), whereas 11.7% and 28.3% of the study participants were moderately and mildly anemic, respectively. Even though the proportion of immediate postpartum anemia among current study participants was relatively low compared to most other studies, it is considered a public health problem according to the WHO classification [32].

The odds of immediate postpartum anemia among postpartum mothers who experienced massive postpartum blood loss were almost five times higher than the odds of anemia among postpartum mothers who did not develop postpartum hemorrhage [AOR = 4.76, 95%CI: (2.44–9.28)]. Similar findings were reported in Debremarkos [5], Saudi Arabia [2], and Tamil Nadu, India [29]. Excessive bleeding after birth decreases the red blood cell component called hemoglobin. In every milliliter of blood loss, a half-milligram of iron will be reduced in the blood [33].

The odds of immediate postpartum anemia were sixfold higher among postpartum mothers who had not taken IFA supplements compared to their counterparts [AOR = 6.19; 95% CI: (2.69,14.22)]. This finding was in agreement with the studies carried out in Debremarkos [5], Uganda, and Pakistan [31, 34]. The possible explanation might be due to the depletion of stored maternal iron since the physiologic requirements of iron during pregnancy and labor are high. Therefore, not taking IFA supplements could reduce the body’s iron stores and result in anemia, even with minimal blood loss during childbirth. The odds of postpartum anemia were two times higher among postnatal mothers whose MUAC measurements were < 23 cm compared to those whose MUAC measurements were ≥ 23 cm [AOR = 2.02, 95% CI: (1.11, 3.68)]. This study was supported by the study done at Debremarkos and Mekelle [5, 24]. The most likely explanation might be iron deficiency anemia, which is usually related to nutritional deficiency. A MUAC measurement of < 23 cm indicates poor muscle mass and lack of adequate energy intake. Hemoglobin concentration and maternal MUAC had a linear relationship, which was also another explanation [35].

Prolonged-second stage labor increases the chance of immediate postpartum anemia occurring almost three times compared to their counterparts [AOR = 2.52, 95% CI: (1.16–5.44)]. This finding was supported by a study done in Indonesia [21]. The possible explanation might be the second stage of labor is prolonged and managements like trial of instrumental delivery, episiotomy, perineal tears, and C/S delivery are expected, which increase the chance of bleeding and the risk of immediate postpartum anemia [5, 36].

### Limitation of the study

There may be recall bias since participants were requested to give information about the past nine months’ “activity of mothers.” Besides, in the postpartum period, after 24 h, there is a possibility of secondary postpartum hemorrhage persisting. Since these women were not eligible to participate in this study, it is possible that the proportion estimation was lower than the actual.

## Conclusion

The proportion of immediate postpartum anemia in Shewarobit health facilities was a significant public health concern as earlier research done in Mekelle and Debre Markos, in Ethiopia. Immediate postpartum anemia was substantially correlated with primary postpartum hemorrhage, not taking IFA supplementation, a prolonged second stage of labor, and MUAC less than 23 cm. By comprehending the local context of anemia in the early postpartum period, this finding may assist concerned bodies and stakeholders in improving women’s health through monitoring, putting preventive measures into place, and sustaining efforts on the identified risk factors of immediate postpartum anemia during pregnancy, labor, and delivery.

## Data Availability

The full data set and other materials pertaining to this research can be obtain from the corresponding author on reasonable request.

## Abbreviations

AOR: Adjusted Odds Ratio
APH: Ante Partum Hemorrhage
CI: Confidence Interval
CBC: Complete Blood Count
COR: Cruds Odds Ratio
DHS: Demographic Health Survey
IAVD: Instrumental Assisted Vaginal Delivery
IFA: Iron and Folic Acid
IPPA: Immediate Post-Partum Anemia
MUAC: Mid Upper Arm Circumference
OR: Odds Ratio
SPSS: Statistical Package for Social Science
SVD: Spontaneous Vaginal Delivery
WHO: World Health Organization
